# Biological and Sociopolitical Sources of Uncertainty in Population Viability Analysis for Endangered Species Recovery Planning

**DOI:** 10.1038/s41598-019-45032-2

**Published:** 2019-07-12

**Authors:** Carlos Carroll, Robert C. Lacy, Richard J. Fredrickson, Daniel J. Rohlf, Sarah A. Hendricks, Michael K. Phillips

**Affiliations:** 1 Klamath Center for Conservation Research, Orleans, CA 95556 USA; 2 Species Conservation Toolkit Initiative, Chicago Zoological Society, Brookfield, IL 60513 USA; 3 Missoula, MT 59812 USA; 4 Earthrise Law Center, Lewis and Clark Law School, Portland, OR 97219 USA; 5 Institute for Bioinformatics and Evolutionary Studies, University of Idaho, Moscow, ID 83844 USA; 6 Turner Endangered Species Fund, 901 Technology Blvd, Bozeman, Montana, 59718 USA

**Keywords:** Population dynamics, Conservation biology

## Abstract

Although population viability analysis (PVA) can be an important tool for strengthening endangered species recovery efforts, the extent to which such analyses remain embedded in the social process of recovery planning is often unrecognized. We analyzed two recovery plans for the Mexican wolf that were developed using similar data and methods but arrived at contrasting conclusions as to appropriate recovery goals or criteria. We found that approximately half of the contrast arose from uncertainty regarding biological data, with the remainder divided between policy-related decisions and mixed biological-policy factors. Contrasts arose from both differences in input parameter values and how parameter uncertainty informed the level of precaution embodied in resulting criteria. Policy-related uncertainty originated from contrasts in thresholds for acceptable risk and disagreement as to how to define endangered species recovery. Rather than turning to PVA to produce politically acceptable definitions of recovery that appear science-based, agencies should clarify the nexus between science and policy elements in their decision processes. The limitations we identify in endangered-species policy and how PVAs are conducted as part of recovery planning must be addressed if PVAs are to fulfill their potential to increase the odds of successful conservation outcomes.

## Introduction

Recovery plans act as blueprints guiding actions that determine whether endangered species survive or perish. Recovery criteria in turn are the goalposts established within each recovery plan which, if met, indicate that a species no longer requires the protection afforded by listing as endangered or threatened^1^. The US Endangered Species Act (ESA; 16 U.S.C. §1531–1540) requires that agencies base their decisions to add or remove taxa from the list of endangered species (and by implication, the recovery criteria on which these decisions depend) solely on biological data rather than economic costs^2^. The “solely” biologically‐based requirement was added to the ESA a decade after its passage due in part to concerns that considering non-biological (e.g., socioeconomic) factors might prompt agencies to declare species recovered at a stage when population size and other factors were still biologically insufficient to ensure their persistence^3^. Lawmakers sought to ensure transparent and evidence‐based recovery planning, with a clear distinction between decisions based on biological data and those based on policy preferences, in order to increase the likelihood of successful conservation outcomes and ensure government accountability to the public^2^. However, this mandate for science‐based criteria is often challenged during real‐world recovery planning processes when potential conservation actions negatively affect influential stakeholders.

Recovery of the gray wolf (*Canis lupus*) and other large mammalian carnivores remains controversial in many nations due to potential impacts on livestock production and the wild ungulate populations hunted by humans^4^. The Mexican wolf (*C*. *l*. *baileyi*), which historically occurred in northern Mexico and the southwestern US, was extirpated from the wild by the 1980s due to such conflicts^5^. Descendants of the seven founders of the captive population were reintroduced onto public lands in the southwestern US beginning in 1998^6^. Whereas earlier gray wolf and Mexican wolf recovery plans used expert judgement to develop recovery criteria, the US Fish and Wildlife Service (henceforth “Service”) has based recent Mexican wolf recovery planning on population viability analysis (PVA), a quantitative tool for systematically eliciting and synthesizing information on factors affecting the demographic and genetic status of threatened species, and determining the influence of these factors on population viability and endangerment^7^.

In 2013, a team of scientists convened by the Service employed PVA to develop draft Mexican wolf recovery criteria^8^. These criteria, which were ultimately shelved after generating opposition from prominent politicians in southwestern US states^9^, proposed that a metapopulation totaling 750 wolves within the US would be necessary for recovery of the subspecies^8,10^. In 2017, a new set of recovery criteria were developed via a PVA conducted with greater involvement by state representatives. (Several of this study’s authors participated in one or both of the recovery teams). When compared to the 2013 effort, these new criteria called for less than half as many wolves (320) inhabiting a smaller portion of the southwestern states^6^. Although the 2013 and 2017 plans propose establishment of additional smaller populations in Mexico, both assume little or no connectivity between US and Mexican populations. Additional criteria addressing genetic inbreeding and other threats also differed substantially between the two plans (SI Table S1).

Because they were developed using similar data and methods but arrived at contrasting conclusions, the 2013 and 2017 recovery criteria provide a unique opportunity to examine how tensions between socioeconomic concerns and the implications of biological data can result in challenges to evidence‐based recovery planning. If rigorous PVA processes for a well‐studied species can produce strongly contrasting criteria, PVA may be limited in its ability to inform this aspect of recovery planning. Alternatively, if contrasting recovery criteria resulted from participants inappropriately distorting conclusions as to how many wolves were necessary for biological recovery, limitations in existing policy and planning processes may need to be addressed. We recognize that recovery planning science is embedded within a sociopolitical process, and that lawmakers have set a high bar by requiring that certain elements of this process be solely science-based. As we detail below, recovery criteria are inevitably informed by values-based decisions, including what level of extinction risk is “acceptable”. Our goal is to establish best practices for conducting PVAs that clearly distinguish science and policy elements and transparently represent available information that can be used to identify the criteria for success in species recovery.

To identify the origins of the contrasts between the two PVAs, we conducted a sensitivity analysis to categorize what proportion of the contrasts in PVA results originated from uncertainty in demographic parameters as opposed to differing interpretations of policies regarding ESA implementation. Based on our results, we propose methods to address identified sources of uncertainty (both biological and sociopolitical) and strengthen the utility of PVA in recovery planning. The lessons from this comparison resonate beyond the US context, for example in the controversy over the appropriate size of the Swedish wolf population^11,12^, because they highlight key aspects of the conceptual framework underpinning endangered species recovery that remain contested and present pitfalls to successful recovery planning in many nations.

## Results and Discussion

The gray wolf is one of the most well‐studied mammalian carnivores, and the wild Mexican wolf population itself has been closely monitored since reintroductions began in 1998^6^. However, our results demonstrate that substantial uncertainty in PVA results should be anticipated when planning for recovery of even a well-studied species. On the one hand, demographic rates of a reintroduced population may differ from those documented for populations in other, ecologically-distinct regions. On the other hand, parameters observed in the reintroduced population during its initial years are not necessarily characteristic of the larger population at the time of recovery which the PVA seeks to predict^13^. The relative weight to assign these two sources of information is one of several decisions that underlie contrasts between the two Mexican wolf PVAs.

**Figure 1:**
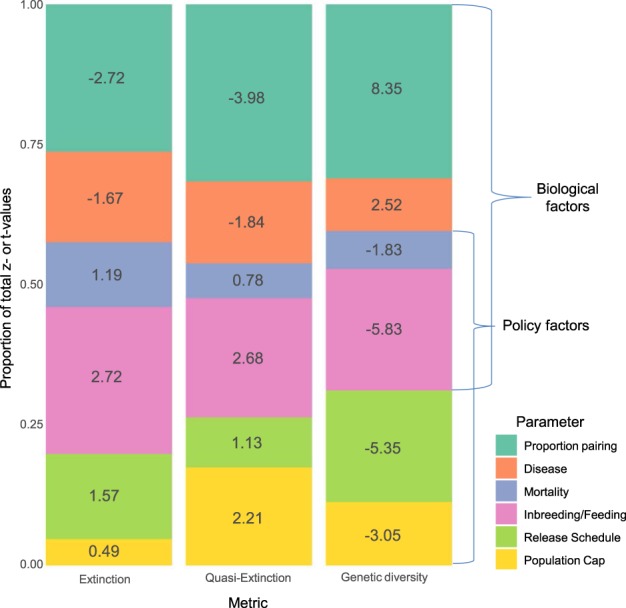
Stacked barplots showing standardized regression coefficients (z- or t- values; values shown within bars of plot) for six variables from regression models predicting contrasts between two population viability analyses (PVA) for the reintroduced US Mexican wolf population in three output metrics (**a**) extinction probability, (**b**) quasi‐ extinction probability, (**c**) genetic diversity). For those variables with positive coefficients, use of parameter values from the 2017 PVA resulted in higher values for the output metric. For those variables with negative coefficients, use of parameter values from the 2017 PVA resulted in lower values for the output metric.

This biological uncertainty in turn can be distinguished from policy‐based uncertainty, which originates from the social process of recovery planning and cannot be reduced by gathering additional data on the species of concern. Our results indicate that about half (40–46%, depending on which PVA output metric was considered) of explained contrast (summed z or t values^14^) between the 2013 and 2017 Mexican wolf recovery criteria was attributed to two parameters we categorized as biological (proportion of females pairing, disease effects) (Fig. 1). One-fifth to one-third (20–31%) of explained contrast was attributed to parameters related to policy (number of releases from captive to wild population, and population cap (the size at which management would aim to keep populations)). Inbreeding and its interaction with the proportion of packs receiving supplemental feeding, as well as adult mortality rate, which we characterized as mixed biological and policy factors as described below, accounted for the remaining 27–38% of explained contrast.

The relative importance of the six factors varied depending on which of three PVA output metrics (extinction probability, quasi‐extinction probability, and genetic diversity) was being predicted (Fig. 1). For extinction probability, proportion of females pairing, inbreeding, and number of releases were the most important factors (highest z value^14^) within the groups of biological, mixed, and policy factors, respectively. For quasi‐extinction probability and genetic diversity, results were similar except that population cap assumed more importance (Fig. 1). Based on the sensitivity analysis results, we make recommendations below for addressing the different types of uncertainty (Table 1).

**Table 1:** Recommendations for addressing uncertainty in population viability analysis (PVA) for endangered species recovery planning.

Category	Recommendation
PVA methods	1. Conduct comprehensive sensitivity analysis;
	2. Avoid underestimation of stochastic threat factors due to field data of limited duration;
	3. Interpret PVA output as illuminating system behavior rather than projecting specific outcomes;
Recovery criteria	4. Base recovery criteria and other conservation goals on those metrics that prove most robust to uncertainty, rather than solely on extinction probability;
	5. Address the implications of data uncertainty via use of values for recovery criteria and conservation goals that incorporate precautionary buffers;
	6. Address uncertainty regarding the future magnitude of specific threats by specifying their alleviation via a quantitative recovery criterion or goal;
Policy development	7. Establish policy guidance specifying appropriate normative thresholds, quantitative frameworks for criteria development, and best practices for determining the composition and operation of recovery teams;
	8. Strengthen scientific integrity policies to require independent peer review of adherence to PVA best practices and substantive response to reviews.

An important advantage of PVA is that it generates not only point estimates of metrics such as mean probability of extinction, but also a distribution of each metric that reflects uncertainties. In the face of uncertainty, sensitivity analysis becomes a key element of a robust PVA. It provides information on the relative influence of uncertainty in each input parameter on predicted population dynamics, but also suggests how much overall confidence we can place in PVA projections, given the aggregate effects of parameter uncertainty^14^. A comprehensive understanding of the implications of uncertainty can inform resilient strategies, i.e. the degree of precautionary buffer necessary in population thresholds and other recovery criteria to ensure the criteria’s adequacy over a range of uncertain parameter values (Table 1, recommendation 1).

### Influence of social structure and unequal reproductive contribution on effective population size

**Figure 2:**
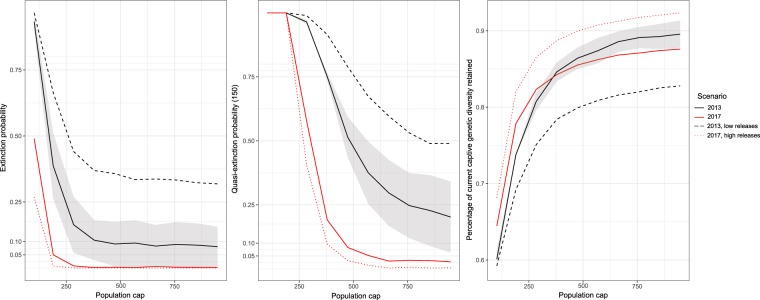
Plots of variation of three population viability analysis (PVA) output metrics (**a**) extinction probability, (**b**) quasi‐ extinction probability, (**c**) genetic diversity) in relationship to population cap and release rate. Gray area indicates range of uncertainty in 2013 PVA results due to alternative functions for proportion of females pairing. “High releases” indicates number of releases proposed in 2013, whereas “low releases” indicates number of releases proposed in 2017.

In a social canid, pack structure results in a substantial proportion of adults being excluded from breeding with consequent effects on genetically effective population size (*N*_*e*_) and rate of accumulation of inbreeding^15,16^. The largest source of variation between the PVA results informing 2013 and 2017 recovery criteria arose from contrasting values for the parameter that represents this factor, termed proportion of adult females pairing^17^. The 2017 PVA’s value (77.6%) was based on the mean of two estimates (68.8%, 86.3%) from the reintroduced Mexican wolf population itself^18^ (see SI Table S2 for parameter values used in 2013 and 2017 PVAs). We reviewed nine published studies and found a mean proportion of females pairing of 68.1% (range 36‐97%, SD 19.4%)(SI Table S3). In some cases, the proportion of adult female wolves pairing in a given year is density dependent, decreasing as wolf numbers increase relative to prey biomass^19^. The 2013 PVA’s parameter value (50%) matched that used in a previous Mexican wolf PVA^20^ but was towards the lower end of the studies we reviewed here (SI Table S3). This suggests that the best‐supported parameter value would be intermediate between those used in the 2013 and 2017 PVAs. However, an unknown proportion of the contrast in pairing parameter values between the 2013 and 2017 model, as well as in the literature, arose from contrasts in the degree to which females without offspring in a particular year were included in the category of potentially reproductive wolves. Results using an alternative sensitivity analysis structure that considered pairs without a litter jointly with unpaired wolves as part of the same sensitivity analysis factor showed reduced importance of the pairing and inbreeding factors and increased importance of policy-related factors (Supplementary Information SI Fig. 1). Carroll *et al*.^10^ also compared results using a fixed parameter value and a density‐dependent value ranging from 30 to 60%, and found this aspect of model structure the fifth most important factor affecting extinction risk predictions (gray area in Fig. 2). This confirms conclusions from previous reviews noting the sensitivity of PVA models to the manner in which density dependence is represented^21^.

The Vortex software incorporates a simplified representation of the stochastic processes that result in pairings between individuals in the wild population^17^. Despite uncertainty surrounding estimation of the pairing parameter, the larger intuitive lesson provided by the PVA results is that minimum viable size for a population with highly unequal reproductive contribution will be greater than would be expected for a species without such dynamics because observed effective population size (*N*_*e*_) will be far below that expected from the census size (*N*) of the population. This effect is strongly evident in data from the wild Mexican wolf population, where 96% of the wolves for which individual genetics are known are descendants of a single superbreeder^22^. Such observations demonstrate that other factors that contribute to non‐random mating can accentuate the effects of the pairing parameter in reducing effective population size.

Because the pairing parameter is highly influential and subject to substantial uncertainty, a resilient recovery strategy might initially incorporate a precautionary buffer in recovery criteria sensitive to this parameter (e.g., minimum population size) while monitoring the wild population for both the reproductive parameter and genetic metrics that reflect its influence (Table 1, recommendation 5). Concluding that recovery criteria are adequate based primarily on the PVA’s point estimates, as was done in the 2017 recovery plan^6^, is an implicitly risk-tolerant approach that is at odds with the ESA’s “policy of institutionalized caution” towards preventing species extinction and advancing recovery (Ariz. Cattle Growers Association v. Salazar, 606 F.3d 1160, 1167 (9th Cir. 2011)).

### Stochastic effects of disease frequency and severity

A strength of Vortex and other PVA simulation models is that they can incorporate effects of infrequent stochastic events such as disease outbreaks. Disease frequency and severity often must be estimated based on data from other populations of the same or similar species, because sufficiently lengthy time series on disease occurrence are unavailable in the early stages of recovery efforts. The 2017 PVA model incorporated a relatively low disease frequency and severity because little is yet known regarding these factors in the wild Mexican wolf population, although high year‐to‐year variation in pup survival^6^ suggests the influence of disease. In contrast, the 2013 PVA model based parameter values for frequency and severity of disease outbreaks on a longer time‐series of data from a population elsewhere in the western US (Table 1, recommendation 2)^23,24^. This contrast between disease parameter values in turn strongly affected predicted extinction and quasi-extinction rates (Fig. 1).

### Influence of mortality rate on viability

Human-caused mortality constituted 81% of Mexican wolf mortalities with known causes from 1998 to 2011, and is the primary threat to persistence of wolf populations globally, particularly where wolves occupy landscapes used by humans and livestock^13,25^. Contrast in mortality parameter values between the 2013 and 2017 PVAs was moderately influential in affecting extinction probability and genetic diversity (Fig. 1). Results of this absolute sensitivity analysis, in which parameters were varied between specific sets of values, differ from previous relative sensitivity analysis results^10^, where mortality was the most influential parameter when all parameters were varied by a fixed percentage.

Because the adequacy of recovery criteria (e.g., minimum population size) is contingent on assumptions regarding future mortality rates, the 2013 draft plan included a criterion requiring that the rate of human‐caused wolf mortality be reduced below the specific level assumed in the PVA before delisting (Table 1, recommendation 6)^8^. By creating an additional recovery criterion based on an uncertain demographic parameter, the 2013 draft plan ensured that a key assumption regarding future threat amelioration was met at the time of delisting. In contrast, the 2017 plan opted against creating a quantitative mortality criterion, in deference to resistance by stakeholders to establishing recovery criteria predicated on changes in human behavior^26^.

### Effects of inbreeding and supplemental feeding on fecundity

Inbreeding depression, the reduced biological fitness that occurs in a population as a result of breeding of related individuals, has been documented in many small populations^27^. The Mexican wolf population currently shows high levels of relatedness equivalent to individuals being as related as full siblings in a non‐inbred population, a level of relatedness similar to that of the Isle Royale wolf population before its recent decline due to genetic issues^28–30^. The level of founder genome equivalents (2.04) is lower than that of any other reintroduced endangered species in North America, except possibly the black‐footed ferret (*Mustela nigripes*)^29,31^. In the Mexican wolf, inbreeding has been found to affect fecundity by increasing the odds that a pair fails to produce any offspring^15^ or by reducing the size of those litters that are produced^31^.

However, stakeholders involved in recovery planning processes often are less familiar with genetic and other stochastic threats (the small‐population paradigm^32^) than they are with the focus of traditional wildlife management on processes controlling dynamics of large populations over short timeframes^33^. For example, a review by several participants in the 2017 planning process states that because deterministic factors “are often larger, more imminent threats” to persistence than are genetic threats, the latter factors should be assigned less importance in the planning process^34^. Similar disagreements as to the importance of genetic threats have typified the controversy over an appropriate size for the Sweden wolf population^11,12^.

The Vortex PVA model was developed in large part to allow more accurate assessment of stochastic threats to small populations^17^. Given appropriate parameter values, Vortex output will reflect the relative impact of deterministic and stochastic factors on population persistence, and the importance that planners should assign to those threats^35^. The contrasting importance assigned to genetic threats in the 2013 and 2017 plans stems in part from the fact that genetic and small population threats can be difficult to estimate directly from the short‐duration datasets available for many species of concern, and must be inferred from data on other species or populations. It is particularly difficult to accurately estimate inbreeding effects based on data from the wild Mexican wolf population because a large proportion (~70%) of wild packs currently receive supplemental feeding that masks deleterious inbreeding effects^6^.

In the Vortex model, reproductive rates are influenced by multiple factors including inbreeding effects, the proportion of the population in which those effects are masked by feeding, as well as age-related variation in fecundity. We found that contrasts in reproductive rates between the 2013 and 2017 PVA were highly influential in explaining contrasts between the two PVA’s results (Fig. 1). Fecundity was generally lower in the 2017 model than in 2013, primarily due to greater age-related variation in fecundity rather than contrast in inbreeding effects, which were partially masked in the 2017 model due to the assumption that many wild packs would receive supplemental feeding.

Over the past decade, scientists and policymakers have debated whether the ESA’s mandate for recovery of self‐sustaining populations should be replaced by an aim to recover species to a “conservation‐reliant” condition in which their persistence remains contingent on long‐term intensive management intervention^36–38^. This contrast in approaches is evident between the 2013 and 2017 Mexican wolf recovery plans. The 2017 plan proposes a long‐term dependence on supplemental feeding of 15% of the wild population to boost demographic rates in the face of elevated inbreeding and human‐caused mortality^6^. We found that, because feeding masked the effects of inbreeding depression, extinction risk was reduced under such feeding strategies. This suggests that the adequacy of the 2017 recovery criteria is contingent on a “conservation‐reliant” approach involving long-term feeding, whereas more ambitious criteria would instead facilitate recovery of populations which do not require such support, a strategy more consistent with the ESA’s mandate. Because feeding tends to occur for the same packs over multiple years, it may also be genetically counterproductive, facilitating the production of highly inbred individuals and accentuating effects of unequal reproductive contribution on effective population size^26^.

### Effects of releases from captivity to the wild population

Because the captive population of Mexican wolves is currently more genetically diverse than is the wild population^39,40^, the number of wolves released from captivity into the wild population has a strong effect on genetic diversity in both PVAs (Fig. 2c). However, in part because the 2017 PVA assumes a level of feeding which masks deleterious inbreeding effects, number of releases does not strongly affect extinction risk in the 2017 PVA (Fig. 2a). The conclusion in the 2017 recovery plan that genetic threats to the Mexican wolf population will have been addressed once 22 wolves have be released from captivity into the US wild population and survived to breeding age is contingent on assumptions regarding feeding effects. This conclusion also provides an example of the limitations of interpreting PVA output as literal predictions.

PVA is best seen as a decision‐support tool for informing effective recovery strategies, rather than as a means to predict the detailed future status of the population^41^. By focusing on the system dynamics revealed by PVA results rather than on point estimates of output metrics, planners can design recovery strategies which are resilient to uncertainty (Table 1, recommendation 3). Because the pedigree of individuals actually released into the wild will not closely match the pedigrees of individuals projected to be released in the simulations, the actual genetic contribution of released wolves is unlikely to closely match results simulated in the PVA model. A resilient recovery strategy would base criteria addressing genetic threats on direct assessment of genetic metrics in the wild population over time rather than the total number of releases completed^28^. Direct assessment of genetic metrics in reintroduced populations is increasingly feasible due to advances in genomics^27,42^.

### Effect of population size on viability

The contrast in the population cap (the management threshold above which removals to reduce population begin) between the 2013 and 2017 PVAs was influential in affecting quasi‐extinction probability and genetic diversity, but had less influence on extinction probability (Fig. 1). We found major contrasts between estimates of extinction probability based on the 2013 and 2017 PVA parameters (Fig. 2a). Our 2013 and 2017 results are qualitatively similar to those of Carroll *et al*.^10^ and Miller^18^, respectively, but show some contrasts with these previously‐published estimates due to several factors. Our results using the 2017 parameters are more optimistic than those reported in Miller^18^ because our model does not remove wolves from the US population to support populations in Mexico. Our results using the 2013 parameters are more pessimistic than those in Carroll *et al*.^10^ because the latter study proposed establishment of two new subpopulations via releases from captivity, which allowed genetic diversity from the captive population to more effectively reduce inbreeding within the metapopulation as a whole than do releases into a single extant population as modeled here. Additionally, the population-cap-related removal rate used here matched that used in Miller^18^, but was more aggressive than that used by Carroll *et al*.^10^.

The ESA qualitatively emphasizes the high degree of protection lawmakers intended to afford to biodiversity. However, the statute does not explicitly define quantitative thresholds for what would constitute an “acceptable” extinction risk for listed species. Recovery criteria thus inevitably are informed by normative decisions such as what level of extinction risk is acceptable^43^. The 2017 PVA used a risk tolerance of 10% in 100 years. When this threshold value was questioned by scientific peer reviewers, the Service justified its use as being within the range of thresholds used in previous recovery plans^26^.

Based on analysis of data^44,45^ for 60 species with quantitative extinction risk thresholds in their recovery plans, we found that thresholds have varied from 1‐10%, but most plans (73%) used a 5% extinction risk threshold. However, justification for this value may be no more than familiarity due to use of 5% as an arbitrary threshold for Type I statistical error. Whether extinction of 117 (5%) of the 2348 species listed under the ESA^46^ is acceptable is inherently a normative decision.

The four recovery plans previous to that for the Mexican wolf that have used a 10% extinction risk threshold include a plan for nine species of cave‐dwelling invertebrates, the white abalone (*Haliotis sorenseni*), the Rio Grande silvery minnow (*Hybognathus amarus*), and the Mariana fruit bat (*Pteropus mariannus mariannus*)^44,45^. The use of a 10% extinction risk tolerance in the 2017 Mexican wolf PVA is unusual when compared with past recovery plans for vertebrate species, especially large mammals. Although the 2013 PVA compared PVA results against an extinction risk threshold of 5% over 100 years, that analysis did not emphasize point estimates of extinction risk, focusing instead on recovering populations that would have >50% probability of meeting or exceeding a quasi-extinction threshold that would prevent the need for relisting as endangered^10^.

Lawmakers may have expected at the time of the ESA’s passage that the agencies would issue regulatory guidance specifying appropriate risk thresholds, limiting their discretion to appropriate levels consistent with the statute (Table 1, recommendation 7)^47,48^. However, to date the relevant agencies (US Fish and Wildlife Service and National Marine Fisheries Service) have chosen to retain maximum discretion to set ad hoc risk thresholds in individual recovery plans, which are then characterized as scientific to avoid the perception that they are defining recovery in a manner that prioritizes policy objectives over science^48^.

### Relationship of quasi‐extinction probability to population size

Early PVA literature focused on estimation of minimum viable population size (MVP)^49^. However, extinction rate and hence MVP is highly dependent on stochastic factors and thus tends to be more sensitive than other PVA output to minor variations in parameter values and model structure. Use of MVP to set both maximum and minimum allowable population size increases the consequences of uncertainty in MVP estimates because the population remains indefinitely at or near this threshold. More recent work suggests that planners, rather than focusing on a single MVP number, should instead use a broader set of PVA metrics to design an effective strategy that allows a population to reach and surpass the stage at which small‐population factors such as genetic inbreeding are important (Table 1, recommendation 4)^41^. Recent reviews and recovery plans have called for use of quasi‐extinction risk thresholds as a more robust alternative to extinction risk^50,51^. Quasi‐extinction analysis can inform criteria for downlisting from endangered to threatened, based on a probability of dropping below a certain population size, e.g. one which might trigger relisting of the taxon as endangered^52^. Reducing the risk of quasi‐extinction may incidentally result in lower extinction thresholds^10^.

In our results, the genetic diversity metric showed much lower, and quasi-extinction probability slightly lower sensitivity to parameter uncertainty than did extinction probability. Quartile coefficients of dispersion for extinction probability, quasi‐extinction probability, and genetic diversity were 1, 0.95, and 0.05, respectively. Genetic diversity results were also less affected by uncertainty due to density-dependent effects (gray area in Fig. 2) than was extinction probability.

### Relationship of genetic diversity to population size and number of releases

The thresholds informing recovery criteria, even if not directly normative as is extinction risk, may nonetheless indirectly embody values‐based decisions. Criteria for addressing genetic threats (i.e., avoiding deleterious levels of inbreeding) provide an example. Genetic recovery goals are typically expressed in relation to the genetic diversity present in the “founder population” (those animals originally taken from the wild to form the captive population), or the genetic diversity held in the captive population at the time the recovery plan is written^53^. For example, the goal for Florida panther recovery was to retain 90% of current genetic diversity for 100 years or longer^54^. The goal of retaining 90% of existing diversity has been widely used^12,27^ because retaining 90% of the original ability to respond to selection is a reasonable target for retaining evolutionary flexibility^42^. Given that that genetic diversity of the Mexican wolf captive population is already greatly depleted due to the small number of founders and the subsequent losses of diversity during generations in captivity, it is important to minimize further loss of genetic variation^28^.

Our results suggest that retaining this level of genetic diversity in the wild population would necessarily involve a large number of initial releases to fully represent the captive population’s diversity within the wild population, followed by steps to allow the wild population to grow significantly larger (in both census size and genetically effective population size) than the captive population, which is limited to the 250–300 individuals that can practically be maintained within the zoo network. In our results, the number of initial releases from the captive to wild population strongly influenced the proportion of genetic diversity retained (Fig. 2c). Given a particular number of initial releases, the population cap parameter then influences the proportion of the initial diversity that is retained by year 100. Influence of population size is strongest at or below the level set in the 2017 plan (379) but larger population caps result in a steadily higher proportion of diversity being retained, with only large populations succeeding in retaining >90% of current diversity.

Rather than using the original founder’s or existing (i.e., 2017) levels of genetic diversity as a baseline, the 2017 plan expressed genetic recovery criteria for the wild population in terms of retaining 90% of the depleted genetic diversity that the captive population will hold at some future time. This lower goal allows the 2017 plan to conclude that smaller population caps and numbers of initial releases are adequate to meet a 90% retention goal. This framework exemplifies a “shifting baseline” approach to setting recovery criteria, a term originating from the recognition in fisheries management that as humanity overexploits fish populations, historical amnesia reduces expectations based on ever‐changing and inappropriate reference points^55^.

Observed heterozygosity in the captive Mexican wolf population is declining at a rate of 0.6‐0.7%/year^39^. The Vortex model projects a slower rate of loss by assuming optimal genetic management of the captive population. Under either the observed or modeled rate of loss, using a shifting baseline as the standard against which recovery is measured is inappropriate because such a depleted condition accentuates rather than alleviates genetic threats, whereas the “ESA was enacted not merely to forestall the extinction of species (i.e., promote a species survival), but to allow a species to recover to the point where it may be delisted.” (Gifford Pinchot Task Force v. U.S. Fish & Wildlife Serv., 378 F.3d 1059, 1070 (9th Cir.)).

### Incorporating resiliency, redundancy and representation in recovery criteria

The conservation principles of resiliency, redundancy and representation (the ‘3R’ criteria) developed by Shaffer and Stein^56^ are widely applied in recovery planning^57^. In essence, the 3R framework states that, to be considered recovered, a species should be present in many large populations arrayed across a range of ecological settings. Redundancy of subpopulations in a metapopulation enhances the viability of each due in part to “spreading of risk”, since episodic threats such as disease outbreaks or long-term trends such as climate change may not affect all subpopulations equally^58,59^. However, the contribution of redundancy to species persistence may be difficult to estimate quantitatively using PVA models because most such models underestimate the effects of rare stochastic events^60–62^. The 2013 plan proposed establishing two additional wild populations within large protected areas in the southwestern US which would form a connected metapopulation together with the current wild population. In contrast, the 2017 plan proposed establishing 1–2 additional small populations on fragmented private landholdings in northern Mexico which would have little or no connectivity with the US population^63^. The appropriate geographic focus of recovery, and questions as to whether habitat quality and protection from persecution in Mexico^64^ were sufficient to allow persistence of populations there, has been a major point of dispute concerning recovery strategies for the Mexican wolf^29,63,65^.

### Inherent challenges of PVA-based recovery planning as a social process

Population viability analysis (PVA) can be an important tool for strengthening endangered species recovery planning by focusing attention on key factors influencing population dynamics and allowing quantitative evaluation and science-informed discussion of alternative recovery strategies^7^. However, the extent to which PVAs remain embedded in the often‐contentious social process of recovery planning can go unrecognized, especially because the ESA requires agencies to portray recovery strategies as science-based. Policy-related uncertainty originates from contrasts in the normative (values‐based) thresholds embodied in recovery criteria and from use of alternative reference points to set non‐normative thresholds. Additional uncertainty arises from contrasts in the broader philosophy of how the ESA should be implemented and what is the most appropriate sociopolitical framework for the recovery planning process. The comparison between the 2013 and 2017 PVAs serves as a cautionary tale that identifies limitations in both existing endangered-species policy and how PVAs are conducted as part of recovery planning, limitations that must be addressed if PVAs are to fulfill their potential to increase the odds of successful conservation outcomes.

The larger social debate over the appropriate scope and extent of efforts to recover endangered species poses a sharp challenge to PVA-informed recovery planning processes. Use of PVA-informed processes to build consensus around recovery planning works best when stakeholders, despite holding differing perspectives and types of knowledge, agree on core objectives of what constitutes species recovery itself. However, not all sectors of U.S. society embrace the ESA’s goal of recovering wild, self-sustaining populations, as evidenced by continued legislative efforts to amend the ESA or increase emphasis on conservation through ongoing human intervention rather than ecosystem restoration^36,37^. This social context often makes it difficult to generate scientifically valid guidance for defining recovery in a manner consistent with the ESA’s requirement that delisting be based “solely” on scientific data. Lack of agreement on a valid endpoint for recovery also makes it difficult to devise an inclusive planning process capable of identifying recovery actions that all or most stakeholders ultimately support.

Addressing uncertainty in the context of disputed core objectives is particularly challenging. Inclusive PVA processes typically seek to elicit PVA parameter values via a science-based consensus process^35^. However, as our analysis shows, substantial uncertainty typically exists regarding demographic parameter values even for well-studied species. If participants’ objectives include arriving at more–protective or less–protective recovery goals, those parties may pursue a strategy of suggesting PVA parameter values the participants know will skew PVA results toward their desired outcome. The criteria developed in the 2017 wolf plan, although purportedly drawn from PVA results, match the wolf population threshold previously negotiated between the FWS and state agencies based primarily on socioeconomic concerns^66^. To produce congruence between PVA output and this negotiated agreement on a politically acceptable wolf population size, the 2017 PVA needed to opt for a suite of parameter values that provides relatively optimistic outcomes in terms of species viability, but runs a higher risk of underpredicting extinction probability. Parameter uncertainty should suggest the need for a precautionary approach to devising criteria, rather than a license to select from within the range of plausible parameter values to give results congruent with policy preferences.

The 2013 and 2017 plans employed very different processes for making the crucial decisions that ultimately determined the plans’ criteria. The 2013 draft plan followed a common recovery team structure strategy also used in the never-completed 2003 recovery planning process. In this model, a group of scientists (primarily species experts but also including social scientists) devises the applicable recovery criteria, and a larger recovery team made up of a diverse spectrum of stakeholders devises the management strategy that will achieve these recovery criteria. In contrast, in 2017 a group of species experts, representatives of state wildlife agencies, and state-level political appointees met to develop the PVA, after which Service staff worked with state representatives to develop the criteria and management strategies to achieve them.

In the 2013 model, scientifically defensible recovery criteria were ultimately not politically acceptable due to FWS’s reluctance to move forward in the face of opposition by powerful stakeholders^9^. In 2017, FWS helped to ensure against a similar outcome by allowing state officials to directly influence development of recovery criteria. However, these criteria may prove legally untenable if they violate the science-only provisions of the ESA. The 2017 process also fell short in terms of inclusivity. While some politically-influential stakeholders played a central role in the process, the Service excluded tribal representatives and civil society organizations that participated on previous recovery teams.

### Increasing transparency and consistency of PVA use in recovery planning

The ESA mandates federal agencies to devise recovery criteria based on biological data rather than socioeconomic costs in order to “halt and reverse the trend toward species extinction, whatever the cost.” (Tenn. Valley Auth. v. Hill, 437 U.S. 153, 184 (1978)). This mandate often conflicts with the perspective of influential stakeholders, such as the state representatives involved in the 2017 wolf PVA, who seek to maximize local tolerance by “balancing” recovery efforts with “social considerations”^34^. This conflict between legal and political considerations is not limited to the US context. Efforts to identify a minimum viable size for the Swedish wolf population, as required by European Union regulations, have also been marked by scientific and political disagreements^11,12^.

In response to a similar controversy involving delisting of the Northern Rocky Mountain gray wolf, the court concluded that “[e]ven if the Service’s solution is pragmatic, or even practical, it is at its heart a political solution that does not comply with the ESA” (Defenders of Wildlife v. Salazar, 729F.Supp.2d 1207; 2010). Frustrated with that court’s decision, regional lawmakers subsequently spurred Congress to pass a rider to a budget bill that removed ESA protections from the Northern Rocky Mountain wolf population, demonstrating the ESA’s “science only” mandate for making delisting decisions is ultimately not immune from political intervention. Federal agencies were no doubt aware of efforts by state representatives involved in the 2017 PVA process to secure similar legislation delisting Mexican wolves (House Resolution 5538, Amendment 78, 113^th^ Congress (2016)).

Other examples of a PVA processes improperly influenced by socioeconomic concerns include recovery planning for Northern Spotted Owls (*Strix occidentalis caurina*), whose conservation involved limits on logging that had significant financial implications for the timber industry of the northwestern US. A 2007 recovery plan developed with strong participation by industry representatives was found to be scientifically flawed by independent peer reviewers^67,68^. This criticism was taken up by politicians from the opposing political party, and the plan was subsequently revised when that party won the presidency in the next federal election^69^. In this example, the process eventually resulted in a more credible outcome, but only through a complex mix of scientific, legal and political checks and balances.

Our results suggest that the contrast between the two Mexican wolf PVAs arose not only from contrasts in the chosen input parameter values but also in how the implications of parameter uncertainty informed the level of precaution embodied in resulting criteria. The Service has claimed that the 2013 and 2017 recovery plans merely represent contrasting visions of recovery, and that opting for the 2017 plan’s less ambitious goals is within the agency’s discretion^26^. However, the history of Mexican wolf recovery planning, during which the Service convened and disbanded three successive recovery teams until they secured a set of “science-based” criteria that was politically acceptable to influential state politicians, is clearly at odds with the intent of lawmakers when they established the ESA’s solely science-based requirement. Such situations in which political decisions are characterized as purely science-based should be of concern because they undermine transparent and evidence-based decision-making. Politically-constrained criteria also have real-world consequences for the Mexican wolf in that they lead the Service to underestimate the number of captive individuals that need to be released into the existing wild population and to forego opportunities to establish new populations in unoccupied suitable habitat.

Although policy reforms alone cannot ensure that biologically-necessary recovery actions occur in the face of opposition by influential stakeholders, they can ensure greater transparency in recovery planning processes. To reduce opportunities for inappropriate political influence, agencies should establish policy guidance and regulations specifying consistent normative thresholds for an acceptable risk of extinction over a given timeframe (Table 1, recommendation 8). Agencies should also specify clear best practices for determining the composition of recovery teams and governing how such teams operate and make decisions^48,70^. Quantitative frameworks for informing criteria development, e.g., the downlisting threshold structure used in several National Marine Fisheries Service recovery plans^52^, should be adopted more broadly across agencies to increase consistency. More rigorous scientific integrity policies, along with independent peer review which evaluates adherence to PVA best practices^70^, can also help guard against scientifically-flawed recovery plans. For example, many of the issues discussed in this review were also raised by peer reviewers of the 2017 draft recovery plan, as well as by scientists involved in the PVA itself^26^, but existing peer review policies allow the Service to finalize a recovery plan without substantively addressing reviewers’ concerns^71,72^.

## Conclusion

The challenges facing efforts to recover large carnivores parallel larger science-policy conflicts surrounding issues such as climate change, where politically-acceptable measures are objectively inadequate to achieve stated policy goals to limit temperature increases below a certain threshold^73^. Ultimately, recovery planning science is embedded within a political process in which scientific information is only one of many influences. As a result of this disconnect between science and politics – and to make decisions appear as the products of science-based decision-making as required by law–federal agencies may turn to PVA as a mechanism to produce politically acceptable definitions of recovery. However, any recovery plan that relies on misrepresentation of scientific data faces long odds in effectively achieving biological recovery. Such a recovery plan also runs the risk of failing to resolve social conflicts, and may be vulnerable legally. If, on the other hand, thoughtfully assembled recovery teams conduct PVAs designed to enhance information available to decision-makers tasked with identifying the criteria for success in species recovery, their results can illuminate key elements of population dynamics and strengthen conservation outcomes even in the face of uncertainties such as those documented in the case of Mexican wolves. Ultimately, the example provide by the contrasting Mexican wolf PVAs can advance the process of clarifying the nexus between science and policy elements in population viability analysis and help to establish safeguards to ensure the scientific integrity of recovery planning processes.

## Methods

### Vortex modeling software

Both the 2013 and 2017 Mexican wolf recovery planning processes employed the Vortex PVA software, an individual‐based population model which simulates the effects of both deterministic forces and demographic, environmental and genetic stochastic events on wildlife populations^17^. Vortex allows planners to incorporate detailed information on the genetic composition and pedigree of existing individuals and project the genetic development of the population over time. However, the software only incorporates a simplified representation of the spatial and behavioral factors influencing the dynamics of real‐world populations.

Vortex simulates a population by stepping through a series of events that describe an annual cycle of a sexually reproducing, diploid organism. Vortex tracks the sex, age, and parentage of each individual in the population as demographic events are simulated. Vortex allows the user to specify the pedigree of the starting population and uses the genetic relationships among founders to derive inbreeding coefficients and other genetic metrics in subsequent simulated generations. The software allows tracking of both demographic metrics (population size, time to extinction) and genetic metrics (heterozygosity, allelic diversity, and inbreeding coefficient)^17^.

The 2013 PVA modified the default Vortex model structure to make it more appropriate for a species such as the wolf with a complex social breeding system, by incorporating into the model the persistent monopolization of breeding opportunities by male and female “alpha” individuals, which reduces genetically effective population size (*N*_*e*_) and thus may enhance inbreeding effects^10^. The 2017 PVA model was adapted from that used in the 2013 process and remained similar in structure to the model described in detail in Carroll *et al*.^10^. While contrasts in habitat modeling methods are also evident between the 2013 and 2017 recovery plans, these contrasts do not affect the non‐spatial Vortex model and are thus not the focus of the analysis.

Data used to parameterize demographic rates in the 2013 and 2017 PVAs were drawn from both the wild Mexican wolf population itself^18,31^ and other western US wolf populations^74^. All simulated populations were started with wolves produced from the existing Mexican wolf pedigree^40^. Although the original 2013 model did not simulate future genetic change in the captive population, we have incorporated that aspect here to allow direct comparison to the 2017 model, which did include such simulations.

In this study, we adapted both the 2013 and 2017 PVA models to simulate dynamics of a single population, in order to focus the analysis on the relative influence of the differing parameter values used in the two PVAs (SI Table S2). Whereas the 2017 plan envisions a single US population, the 2013 draft plan proposed establishment of a metapopulation of 3 US subpopulations connected by dispersal. When connectivity between the US and Mexican populations was modeled as one scenario during the 2017 PVA, Mexico acted as a population sink which reduced the viability of the US population^18^. Although simulation of the dynamics of a single population accurately represents the primary processes governing viability in both the 2013 and 2017 PVAs, we describe in Results above where this structure causes results to differ from previous‐published viability projections^10,18^.

### Structure of sensitivity analysis

We used a sensitivity analysis to partition the source of the contrasting results (and hence contrasting criteria) in the 2013 and 2017 PVAs between six key parameters in the model. We included in the sensitivity analysis the four most influential parameters from previous sensitivity analysis^10^, as well as disease effects and a sixth parameter not previously part of sensitivity analyses: the number of wolves released from the captive to wild population. We analyzed inbreeding effects jointly with the effect of supplemental feeding because the reproductive rate function in the Vortex model combined both effects. We use an absolute sensitivity analysis here, based on a pre‐determined set of parameter values, rather than a relative sensitivity analysis which varies all parameter values by a fixed percentage^10^.

We categorized the six factors as biological (proportion of females pairing, disease effects), policy-related (number of releases from captive to wild population, population cap (the size at which management would aim to keep populations)), or mixed biological and policy-related (inbreeding and its interaction with the proportion of packs receiving supplemental feeding, adult mortality rate). Biological and policy‐related variables can be distinguished by the fact that additional data may reduce uncertainty for biological variables, but not for policy‐related variables. Further details on the six parameters are provided in Supplementary Information SI Text 1.

To determine the sensitivity of the model projections to uncertainty, we performed 1000 iterations of 100 years for each factorial combination of the alternative values for the six parameters used in 2013 and 2017. We generated regression models predicting three Vortex output metrics (extinction probability, quasi‐extinction probability, and genetic diversity) from the six parameters (with sample unit being the scenario; *n* = 128). The quasi‐extinction threshold was set at 150 individuals. Logistic regression was used for the binary response metrics (extinction and quasi‐ extinction) and linear regression for genetic diversity. We also measured the quartile coefficient of dispersion for the three output metrics across the factorial scenarios to assess relative sensitivity of alternative metrics to parameter uncertainty.

The standardized regression coefficients (z values) from the logistic regression models and the t values from the linear regression model were then used to assess the relative proportion each of the six factors contributed to the total explained variation between results of the two PVAs. Standardized regression coefficients, generated by dividing a regression coefficient by its standard error, are unitless values whose magnitude indicates the relative importance of a parameter in the model^10,14^. Because the magnitude z-values cannot be compared directly between models for the three different output metrics, we compared what proportion the z-value for a factor represented of the total z- or t-values for each model (Fig. 1).

### Effect of contrasting normative thresholds

Our primary sensitivity analysis addressed the relative effects of alternate parameter values used in the two PVAs. We also addressed how the 2013 and 2017 parameter values affected extinction and quasi-extinction probability and genetic diversity retention at a range of 10 population cap values and two release rates. We also measured the proportion of genetic diversity retained as a proportion of the GD of the current captive population.

To provide context for the discussion of the two PVAs use of alternate normative risk thresholds, we summarized the range and probability distribution of threshold values used in previous recovery plans, based on data from two recent comprehensive reviews^44,45^ which comprised data from recovery plans from 1249 species dating from 1979 to 2012, of which recovery criteria with quantitative risk thresholds existed for 60 species.

## Supplementary information

Supplementary Information

## Data Availability

The datasets analyzed during the current study are available in the zenodo.org repository at 10.5281/zenodo.3239461.
