# Supplementary material for: Biological and Sociopolitical Sources of Uncertainty in Population Viability Analysis for Endangered Species Recovery Planning
Source: Sci Rep. 2019 Jul 12;9:10130. doi: 10.1038/s41598-019-45032-2 (PMC6626004; doi:10.1038/s41598-019-45032-2)
Supplement: Supplementary file 1 — Supplementary Information [file 41598_2019_45032_MOESM1_ESM.pdf]

Supplementary Material for 'Biological and sociopolitical sources of uncertainty in population viability analysis for endangered species recovery planning'

Carlos Carroll[1]*, Robert C. Lacy[2], Richard J. Fredrickson[3], Daniel J. Rohlf[4], Sarah A. Hendricks[5], Michael K. Phillips[6].

1. Klamath Center for Conservation Research, Orleans, CA 95556 USA

2. Species Conservation Toolkit Initiative, Chicago Zoological Society, Brookfield, IL 60513 USA ORCID: 0000-0002-8348-623

3. Missoula, MT 59812, U.S.A. ORCID 0000-0002-1356-3713

4. Earthrise Law Center, Lewis and Clark Law School, Portland, OR 97219, U.S.A.

5. Institute for Bioinformatics and Evolutionary Studies, University of Idaho, Moscow, ID 83844, USA ORCID: 0000-0001-9571-9461

6. Turner Endangered Species Fund, 901 Technology Blvd, Bozeman, Montana 59718 USA.

* carlos@klamathconservation.org

Table S1. Recovery criteria proposed in 2017 and draft 2013 Mexican wolf recovery plans.

Type of criteria	2013 draft criteria	2017 criteria
1. **Population size and number and metapopulation size**	A metapopulation consisting of a minimum of 3 primary core populations in the wild, each with a census population size of at least 200 individuals, and a total metapopulation size of at least 750 individuals.	Mexican Wolf experimental Population Area (MWEPA; the US population) average population abundance is greater than or equal to 320, and Northern Sierra Madre Occidental (Mexico) average population abundance is greater than or equal to 170.
**2. Population trend**	Population trend in each of the 3 primary core populations has a high probability (80% confidence) of being stable or increasing over 8 years, based on a statistically reliable monitoring effort.	Stated population abundance is maintained or exceeded over 8 consecutive years.
3. **Population connectivity (including releases from captive to wild population)**	Immigration into each of the 3 primary core populations via natural dispersal at a rate of at least 1 genetically effective migrant every generation, averaged over a period of 8 successive years, as measured by a statistically reliable monitoring effort. A genetically effective migrant is defined as a wolf that breeds in a non-natal population and produces at least 1 pup that survives to at least December 31 of the year of its birth.	Gene diversity available from the captive population has been incorporated into the MWEPA through scheduled releases of a sufficient number of wolves to result in 22 released Mexican wolves surviving to breeding age in the MWEPA, and 37 released Mexican wolves surviving to breeding age in the northern Sierra Madre Occidental.

4. **Amelioration of human-caused losses (HCL)**	The estimated annual rate of human caused losses averaged over an 8-year period is less than 20% as measured by a statistically reliable monitoring effort. This is the greatest rate of anthropogenic mortality and removal that a Mexican wolf population could have and still be expected to have an approximately 75% or greater chance of being stable or increasing.	None.
5. **Post-delisting monitoring**	To monitor the continued stability of the recovered Mexican wolf, a post-delisting monitoring plan has been developed and is ready for implementation within the affected states as required in section 4(g)(1) of the ESA.	None.
6. **Regulatory mechanisms**	State management plans and adequate post-delisting regulatory protection and capacity confirmed. Components of an adequate plan will include assurances that: (1) the natural dispersal rate required for delisting is not precluded by HCL; and, (2) management targets for population size are sufficiently large relative to delisting criteria and HCL rates are sufficiently low to ensure that there is no greater than a 10% chance that the Mexican wolf will fall below the recovery criteria within a 10-year period.	Effective State and Tribal regulations are in place in the MWEPA in those areas necessary for recovery to ensure that killing of Mexican wolves is prohibited or regulated such that viable populations of wolves can be maintained. In addition, Mexico has a proven track record protecting Mexican wolves. Based on these protections, Mexican wolves are highly unlikely to need the protection of the ESA again.

Table S2. Parameter values from 2013 and 2017 Mexican wolf PVAs compared in sensitivity analysis. See Lacy & Pollak 2012 for details on Vortex parameter coding, and Carroll et al. (2014) and Miller (2017) respectively for more details on parameterization of 2013 and 2017 PVAs.

1) Proportion of females pairing (see text regarding contrasts in definition of this parameter between the 2013 and 2017 PVAs)

2013: non-density-dependent function: 0.50

density-dependent function: MAX((0.60-((0.60-0.30)*((N/425)^2)));0.30), where N is population size.

2017: 0.776

2) Disease frequency and severity

2013: 20%/year probability of reduction in pup survival by 80% and reduction in adult survival by 5%

2017: 15%/year probability of reduction in pup survival by 65% and reduction in adult survival by 5%

3) Annual adult mortality

2013: 22.9%

2017: 24.9%

4) Inbreeding and supplemental feeding effects

a) Inbreeding

2013: i) probability of no litter: modeled within litter size function

ii) litter size: (A<10)*(4.8761 - (8.2327*KIN))

2017:

i) probability of no litter: 100*(1-((A<4)*((EXP(1.266+1.819-(8.255*KIN)))/(1+(EXP(1.266+1.819-(8.255*KIN)))))+((A>3)AND(A<9))*((EXP(1.266+2.2645-(8.255*KIN)))/(1+(EXP(1.266+2.2645-(8.255*KIN)))))+((A>8)*((EXP(1.266-(8.255*KIN)))/(1+(EXP(1.266-(8.255*KIN)))))))

where A is age of dam and KIN is kinship between parents.

ii) litter size: IF(RAND<PROPORTIONPACKSFED;(EXP(1.0937+0.49408+((0.09685*((DAM19-5.292)/2.217))+((-0.12114)*((DAM19-5.292)/2.217)^2))));(EXP(1.0937+((0.09685*((DAM19-5.292)/2.217))+((-0.12114)*((DAM19-5.292)/2.217)^2)))))), where DAM19 is age of dam.

b) Proportion packs supplementally fed:

2013: 0

2017: ((Y<6)*0.7)+(((Y>5)AND(Y<20))*[IF((NYEAR5<97);(0.7-(0.0393*(Y-5)));(MAX[(0.7-((0.000405*(NYEAR5))*(Y-5)));0.15]))])+((Y>19)*0.15)

5) Number of initial releases

2013: 20 pairs with pups (totaling 40 adults and 60 pups)

2017: 14 pairs with pups (totaling 28 adults and 42 pups)

6) Population cap

2013: 379

2017: 758

Table S3. Review of previous estimates of proportion of females pairing in gray wolves. References are listed after SI Supplemental Methods section above.

% Females breeding	Measure	Prey	Wolves	Location	Source
97	Proestrus, estrus, or pregnant	UBI[1] 180 – 390	Central AK: wolves reduced 61% before study; annual 38 – 43% removals.	Central & East central AK	Boertje & Stephenson 1992
96	Proestrus, estrus, or pregnant	UBI 500 - 850	"scarce" due to federal control; increasing in 1960's	Central & East central AK	Boertje & Stephenson 1992
67	Pigmentation or enlargement of teats	Moose density 0.8 / km^2	11 to 20 wolves/1,000 km2	Kenai National Wildlife Refuge, AK	Peterson et al. 1984
66	Proestrus, estrus, or pregnant	UBI 96 - 105	Lightly harvested (16% removed); "nutritionally limited" for at least 3 years prior.	Central & East central AK	Boertje & Stephenson 1992
59	"adult" females	unknown	unknown	Ontario	Pimlott et al. 1969
58	Placental scars	Not stated	Population recolonized over last four decades, and "…subject to varying degrees of illegal harvest, government depredation control, and for the 3 years of sampling, public harvest."	Throughout MN wolf range	Mech et al. 2016
57	Presence of corpora lutea, corpora albicantia, fetuses, or placental scars	"Primary prey species appeared to be readily available."	"Increasing and stabilizing" over the three years of the study.	Keewatin District, NWT, Canada	Hillis and Mallory 1996
54 - 100	Intensive searching for pups	Elk declining 2006 – 2012, increasing 2013 – 2016; Bison increasing 2006 -2014	Ranged from 30 – 77 wolves.<br>Increased 2006 – 2008; declined 2009; stable 2010 – 2016.	Northern range, Yellowstone National Park	D. Smith unpublished data
36	Nipple size	Not stated	Legally protected during most of study period	Superior National Forest, MN	Mech et al. 2016

Figure S1. Stacked barplots showing standardized regression coefficients (z- or t- values; values shown within bars of plot) for six variables from regression models predicting contrasts between two population viability analyses (PVA) for the reintroduced US Mexican wolf population in three output metrics (a) extinction probability, b) quasi- extinction probability, c) genetic diversity). Compared to Fig. 1 in the main article, this figure is based on a sensitivity analysis using an alternative structure which excluded pairs with zero size litters from the "proportion of packs pairing" estimate. 500 iterations of 100 years each were completed for each of the 128 scenarios in the sensitivity analysis.

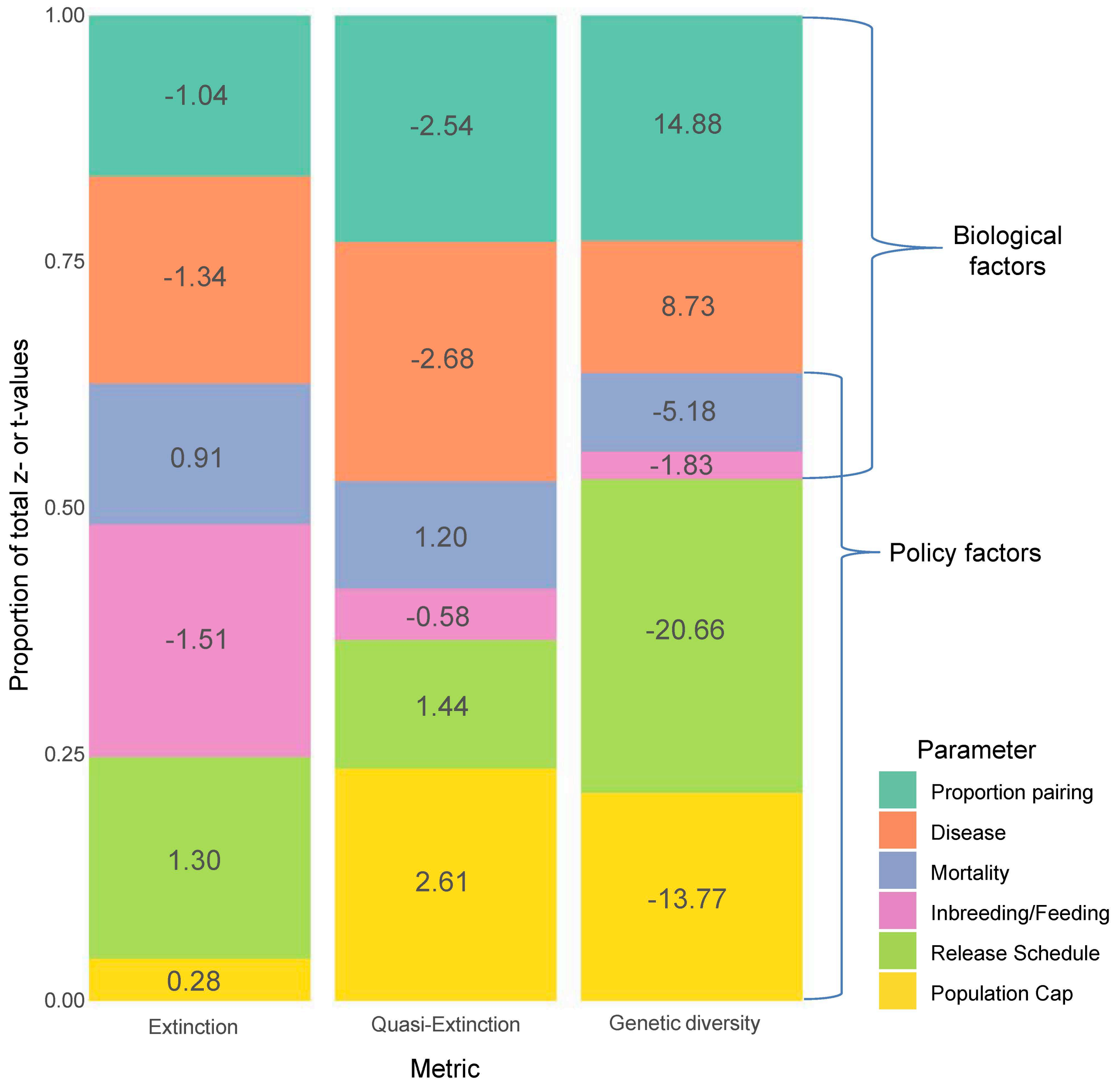

SI Text S1. Supplementary Methods

Proportion of females pairing

Carroll *et al.* [1] found that the proportion of adult females pairing was the second most important parameter affecting extinction risk among simulated populations of Mexican wolves. Higher values for this parameter result in a larger ratio of effective population size to census size and hence reduced rates of inbreeding accumulation.

The proportion of adult females pairing is often difficult to estimate in wild wolf populations. Available data suggest that this proportion may be determined by the density of wolves (which is typically smaller in heavily exploited populations) as well as prey abundance. When wolf populations are at high densities, or at high densities relative to prey populations, wolves may form larger packs in which fewer females breed each year, or females may become nutritionally stressed, reducing the proportion of females that breed [2]. The current wild Mexican wolf population would be expected to have a high rate of females pairing because the population is still in an initial expansion phase from reintroduction, and mortality rate until 2008 was similar to that of a heavily exploited population [3]. These factors would tend to create small pack sizes and opportunities for almost all adult females to breed. This proportion would be expected to decrease as mortality rates are reduced and population density increases, as is assumed to occur before the population can be delisted.

Previous Mexican wolf PVAs have set the proportion of adult females pairing at 50-60% [4,5], whereas wolf PVAs in other regions have used values ranging from 35-57% [6,7]). In this study, we assessed empirical support for alternative parameter values for proportion of females pairing by summarizing the results from nine published studies which reported this parameter (SI Table S2). In replicating the 2013 PVA, we followed Carroll *et al.* [1] in averaging results based on two alternate values: a fixed value of 50%, and a density-dependent function in which the rate dropped from 60% to 30% as population density increased (Table S2). The 2017 PVA used a fixed parameter value of 77.6% based on rates observed in the Mexican wolf population since reintroduction [8]. We also evaluated results from an alternative sensitivity analysis structure which excluded pairs with zero-size litters from the pairing pool in a particular year by grouping together as a single factor in the sensitivity analysis the function governing the proportion of zero-size litters and the function governing the proportion of females pairing.

Disease frequency and severity

Distemper has been detected in the Mexican wolf wild population [9], and a wide variation

in pup count has been observed in successive years (2016; 50 of 113, 2017; 26 of 114) [3] as is consistent with disease effects. Data from other inbred wolf populations such as at Isle Royale suggests that inbreeding depression may make wolves more susceptible to disease and other stochastic threats [10]. The 2013 PVA parameterized episodic threats based on data from the Yellowstone wolf population which showed distemper outbreaks “as often as every 2–5 years”, affecting primarily fecundity rather than survival [11,12]. The 2013 PVA estimated that disease outbreaks occur on average every 5 years, and that in a year with a disease outbreak, fecundity would be reduced by 80%, and survival of all age classes would be reduced by 5% [1]. The 2017 PVA in contrast assumed that disease outbreaks occur on average every 6.7 years, and that in a year with a disease outbreak, pup survival would be reduced by 65%, and survival of all other age classes would be reduced by 5%.

<u>Adult mortality</u>

Adult mortality rate is the most important parameter affecting extinction risk in simulated populations of Mexican wolves [1]. The Mexican wolf population may be especially sensitive to fluctuations in human- caused mortality rates because fecundity has been negatively affected by inbreeding depression [13]. High mortality and removal rates restricted population growth in the first decade after reintroduction [3]. After 2009, restrictions on management removals and increased efforts to reduce livestock depredation (e.g., through supplemental feeding) reduced mortality rates to 18.9%/year, allowing the population to increase from 42 to 114 individuals [3].

However, if recent low mortality rates are contingent on efforts such as supplemental feeding which will not be characteristic of the larger population subsequent to delisting, higher values for this mortality parameter may be more appropriate for use in a PVA. Carroll *et al.* [1] used a base adult mortality rate of 22.9%/year, derived from the wolf population in the Greater Yellowstone Ecosystem (GYE) [14] because they proposed that GYE mortality rates represent a plausible goal for mortality rates after recovery actions are implemented but before delisting. The base 2017 PVA scenario used a slightly higher adult mortality rate (24.9%/year), similar to that experienced by wolves in the Northern Rocky Mountains as a whole prior to delisting [14].

<u>Inbreeding and supplemental feeding effects</u>

Inbreeding depression is a major threat to persistence and recovery of the Mexican wolf [15], and was the fourth most important parameter affecting extinction risk among simulated populations of Mexican wolves [1]. Deriving estimates of inbreeding depression from a relatively small population can be complicated by a number of factors. The genetic relationships and level of inbreeding in the seven founders of the Mexican wolf population were unknown, so pedigree-

based inbreeding estimates could be inaccurate.

Given that only two founder genome equivalents remain in the wild population (equivalent to that expected for full siblings), it may already be fixed for a number of deleterious alleles. In this case there would be no evidence of inbreeding depression because virtually all individuals would have detrimental genotypes. Because effects of inbreeding are often environmentally dependent, supplemental feeding of the majority of the wild population, which occurred starting in 2009 [3], would be expected to mask inbreeding effects and allow pups that would otherwise be compromised by inbreeding to survive [16].

The 2013 PVA modeled inbreeding depression as affecting litter size, based on analysis of data from the wild Mexican wolf population prior to large- scale supplemental feeding [13]. In contrast, the PVA underlying the 2017 Mexican wolf recovery plan analyzed the effect of inbreeding on litter size using a data from both fed and unfed packs. This analysis found no significant effects of inbreeding on litter size, and as a result, the 2017 PVA incorporates inbreeding effects on the probability of producing a litter, but not as an influence on litter size [8].

The high proportion of packs (~70%; [3]) currently receiving supplemental feeding has resulted, and will in the near future continue to result in increased population growth rate in the wild population. However, while feeding masks inbreeding depression, inbreeding effects (e.g., reduced litter size and increased mortality) will become apparent at the time that feeding is discontinued or reduced. Therefore, assumptions concerning long-term prospects for continued feeding of the wild population have large implications for PVA projections. The 2017 PVA assumed that high rates of feeding (70% of packs) would occur during the initial phase of recovery, and that lower rates of feeding (15% of packs) would continue throughout the simulation. The 2013 PVA assumed no supplemental feeding would occur.

Number of initial releases

Because the genetic diversity of the captive Mexican wolf population is currently greater than that in the wild population [17], releases from the captive to the wild population are an effective means of addressing genetic threats. The 2013 PVA assumed that two new wild subpopulations would be founded from the captive population, which required release of 20 pairs with 3 pups each over a period of 5 years. The 2017 PVA assumed for its base scenario that a total of 14 pairs with 3 pups each are released over a period of 16 years.

Population cap

The 2017 recovery plan proposed to establish a population ceiling above the MVP

estimated in the PVA. Given this buffer, it was proposed that the population could be prevented from exceeding this cap via capture or killing without risk of affecting persistence of the population. The 2017 PVA assumed a population threshold of 379 (18% higher than the delisting threshold of 320) after which 100% of "excess" wolves would be killed each year. The 2013 PVA set the removal threshold at the delisting threshold of 750, after which threshold 12.5% of excess wolves were modeled as being killed each year. For the purposes of this sensitivity analysis, we revised the 2013 parameter to make it more comparable to the 2017 structure: the population threshold was set at 758 (twice that used in the 2017 PVA) and removal rate was set at 100%.

REFERENCES

1 Carroll, C., Fredrickson, R. J. & Lacy, R. C. Developing Metapopulation Connectivity Criteria from Genetic and Habitat Data to Recover the Endangered Mexican Wolf. *Conservation Biology* **28**, 76-86, doi:10.1111/cobi.12156 (2014).

2 Boertje, R. & Stephenson, R. Effects of ungulate availability on wolf reproductive potential in Alaska. *Canadian Journal of Zoology* **70**, 2441-2443 (1992).

3 Service, U. F. a. W. Mexican Wolf Recovery Plan, First Revision. *US Fish and Wildlife Service, Albuquerque, New Mexico* (2017).

4 Seal, U. Mexican wolf population viability assessment: Review draft report of workshop. 22-24 October 1990. *Sponsored by International Union for Conservation of Nature, Conservation Breeding Specialist Group. Fossil Rim Wildlife Center, Glen Rose, Texas, USA* (1990).

5 Nature, I. U. f. C. o. Mexican wolf population viability analysis draft report. *Conservation Breeding Specialist Group, Apple Valley, Minnesota, USA.* (1996).

6 vonHoldt, B. M. *et al.* A novel assessment of population structure and gene flow in grey wolf populations of the Northern Rocky Mountains of the United States. *Molecular ecology* **19**, 4412-4427, doi:10.1111/j.1365-294X.2010.04769.x (2010).

7 Ewins, P., de Almeida, M., Miller, P. & Byers, O. Population and habitat viability assessment workshop for the wolves of Algonquin Park: Final report. *IUCN/SSC Conservation Breeding Specialist Group, Apple Valley, Minnesota* (2000).

8 Miller, P. Population viability analysis for the Mexican wolf (Canis lupus baileyi): Integrating wild and captive populations in a metapopulation risk assessment model for recovery planning. *Prepared for US Fish and Wildlife Service, Albuquerque, New Mexico* (2017).

9 Hedrick, P. W., Lee, R. N. & Buchanan, C. Canine parvovirus enteritis, canine distemper, and major histocompatibility complex genetic variation in Mexican wolves. *Journal of Wildlife Diseases* **39**, 909-913 (2003).

10 Hedrick, P. W., Peterson, R. O., Vucetich, L. M., Adams, J. R. & Vucetich, J. A. Genetic rescue in Isle Royale wolves: genetic analysis and the collapse of the population. *Conservation Genetics* **15**, 1111-1121, doi:10.1007/s10592-014-0604-1 (2014).

11 Almberg, E. S., Mech, L. D., Smith, D. W., Sheldon, J. W. & Crabtree, R. L. A serological survey of infectious disease in Yellowstone National Park's canid community. *PloS one* **4**, e7042, doi:10.1371/journal.pone.0007042 (2009).

12 Almberg, E. S., Cross, P. C. & Smith, D. W. Persistence of canine distemper virus in the Greater Yellowstone Ecosystem's carnivore community. *Ecological Applications* **20**, 2058-2074 (2010).

13 Fredrickson, R. J., Siminski, P., Woolf, M. & Hedrick, P. W. Genetic rescue and inbreeding depression in Mexican wolves. *Proceedings. Biological sciences / The Royal Society* **274**, 2365-2371, doi:10.1098/rspb.2007.0785 (2007).

14 Smith, D. W. *et al.* Survival of Colonizing Wolves in the Northern Rocky Mountains of the United States, 1982–2004. *Journal of Wildlife Management* **74**, 620-634, doi:10.2193/2008-584 (2010).

15 Hedrick, P. Genetics and recovery goals for Mexican wolves. *Biological Conservation* **206**, 210-211, doi:http://dx.doi.org/10.1016/j.biocon.2016.12.033 (2017).

16 Armbruster, P. & Reed, D. Inbreeding depression in benign and stressful environments. *Heredity* **95**, 235 (2005).

17 Siminski, P. & Spevak., E. Population analysis and breeding and transfer plan: Mexican wolf Species Survival Plan yellow program. *Association of Zoos and Aquariums, Silver Spring, Maryland. 89pp* (2017).

18 Mech, L. D., Barber-Meyer, S. M. & Erb, J. Wolf (Canis lupus) Generation Time and Proportion of Current Breeding Females by Age. *PloS one* **11**, e0156682, doi:10.1371/journal.pone.0156682 (2016).

19 Peterson, R. O., Woolington, J. D. & Bailey, T. N. Wolves of the Kenai peninsula, Alaska. *Wildlife Monographs*, 3-52 (1984).

20 Hillis, T. L. & Mallory, F. F. Fetal development in wolves, Canis lupus, of the Keewatin District, Northwest Territories, Canada. *Canadian journal of zoology* **74**, 2211-2218 (1996).

21 Pimlott, D.H., Shannon, J.A., & Kolenosky, G.B.. The ecology of the timber wolf in Algonquin Provincial Park, Ontario. Research Report (Wildlife), no. 87. Toronto: Ontario Department of Lands and Forests 1–92 (1969).
